# Staff knowledge, attitudes and confidence levels for fall preventions in older person long-term care facilities: a cross-sectional study

**DOI:** 10.1186/s12877-023-04323-0

**Published:** 2023-09-25

**Authors:** Neah Albasha, Ruth McCullagh, Nicola Cornally, Suzanne Timmons

**Affiliations:** 1 Center for Gerontology and Rehabilitation, School of Medicine, St Finbarr’s Hospital, University College Cork, Block 13, Douglas Road, The Bungalow, Cork, Ireland; 2 Rehabilitation Department, College of Health and Rehabilitation Sciences, Princess Nourah bint Abdulrahman University, Riyadh, Saudi Arabia; 3 Discipline of Physiotherapy, School of Clinical Therapies, University College Cork, Cork, Ireland; 4 School of Nursing and Midwifery, University College Cork, Cork, Ireland

**Keywords:** Fall prevention, Older person, Long-term care, Staff, Knowledge, Attitude, Confidence

## Abstract

**Background:**

Falls are the most common health problem affecting older people in long-term care facilities (LTCFs), with well-recognised adverse psychological and physical resident outcomes, and high staff burden and financial cost. LTCF staff knowledge and skills can play a vital role in providing and promoting fall prevention care.

**Methods:**

A descriptive cross-sectional survey study was conducted across 13 LTCF sites in the Southwest of Ireland; a sampling frame facilitated inclusion of a range of provider types and facility sizes. An existing questionnaire, based on fall prevention guidance, and examining staff knowledge, skills and attitudes, was distributed in physical and online formats.

**Result:**

The response rate was 15% (n = 155), predominantly healthcare assistants, staff nurses and senior nurses. Almost 90% expressed high confidence levels for delivering fall prevention interventions and being aware of how falls affect LTCFs. However, over half underestimated the fall rate in LTCFs, and only 60% had adequate knowledge. Longer experience in working with older people in healthcare services was associated with greater knowledge (p = .001) and confidence in fall prevention interventions (p = .01), while senior nurses had more knowledge than others (p = .01). LTCF staff had lowest knowledge about “identification systems for residents at high risk of falling”, “keeping confused residents near nursing stations”, “the effect of using antipsychotic medicine on falls”, “using a toileting regimen” and “staff responsibility regarding fall prevention efforts”. Despite their knowledge gaps, nearly 50% thought they had enough fall prevention training; their main preference for any further fall education training was face-to-face education.

**Conclusion:**

The results, with the caveat of a low response rate, show the need for interdisciplinary fall prevention training that is tailored to both the perceived learning needs and actual knowledge gap of LTCF staff and their preferences for learning delivery, as part of an overall approach to reducing fall-related adverse outcomes.

**Supplementary Information:**

The online version contains supplementary material available at 10.1186/s12877-023-04323-0.

## Background

One of the most serious health problems affecting older people is falls, being associated with significant mortality and morbidity [1]. The World Health Organisation (WHO) estimates that falls are the second cause of death globally, impacting the lives of 684,000 people every year [2]. Additionally, it was also reported in 2015 that falls account for between 23 and 40% of fatal injuries in the elderly [3]. In long-term care facilities (LTCFs), approximately half of older residents experience more than one fall per year [4], which is three times higher than the rate in community-dwelling older people [5, 6]. According to estimates, each bed in an LTCF has an average of 1.7 falls per year, with 10 to 25% of those falls resulting in a fracture or hospitalisation, as opposed to community-based falls, where 5% are associated with fractures and hospitalisation [5–7]. This disproportionate fall and significant injury rate is due to the specific features of disability, frailty, comorbidity and decreased functional capability among the majority of LTCF residents [7, 8].

Falls often have adverse physical and psychological consequences. Falling causes physical injuries in one-third of older residents, with hip fractures being the most prevalent, occurring in 3–5% of cases yearly [3]. The psychological effects of falls on residents include depression, fear of falling, loss of confidence and a decline in quality of life [3, 9]. Falls also cause an economic burden on the healthcare system through lengthy hospitalisation. According to the National Institute for Clinical Excellence (NICE), the yearly costs of falls and fall-related fractures are 2.3 and 1.7 billion pounds sterling, respectively [1].

Several factors contribute to falls, and these factors frequently interact, making falls multi-faceted. Intrinsic factors include: ageing; impairments in balance, mobility and vision; cognitive impairment; chronic disease, etc., and also psychological issues such as fear of falling and low self-efficacy [10, 11]. Extrinsic fall risk factors involve environmental hazards and medication [10–12]. Fall prevention is a significant clinical quality indicator in healthcare facilities, and one of the core components of patient safety [13]. Healthcare professionals need to work in a proactive and positive way to prevent falls for older people; promoting and maintaining an enhanced quality of care [14, 15]. The multidisciplinary team (MDT) provides a variety of fall prevention approaches, whether individual or multi-factorial programmes, delivered by physiotherapists, geriatricians, nurses and other healthcare professionals [16]. Some multi-factorial interventions can help people who have fallen or are at high risk of falling [17], but need to be organised and coordinated across professions, and staff must be engaged and have skills to conduct assessments and implement fall prevention activities [7, 18].

Fall prevention presents a major challenge for staff in LTCFs. Limited knowledge and skills have been noted as a principal barrier, affecting their understanding of fall risk factors and their ability to engage in fall prevention activities [19–22]. Staff attitudes to falls in LTCFs and their ability to control falls have also been identified as barriers [19]; an attitude is defined here as follows: “a relatively enduring and general evaluation of an object, person, group, issue, or concept on a dimension ranging from negative to positive. Attitudes provide summary evaluations of target objects and are often assumed to be derived from specific beliefs, emotions, and past behaviours associated with those objects” [23]. It is frequently perceived that falls are inevitable and are a highly serious problem in LTCFs. Facilities have been perceived to lack fall prevention strategies, which adversely affected patient safety [24–26]. However this is amenable to change via ongoing professional development aiming to improve fall prevention awareness, confidence and interest [19, 20, 27, 28].

Education has been used as a single intervention or as part of multi-factorial intervention programmes for fall prevention [5]. In many systematic reviews (SRs) and meta-analyses (MAs) in LTCFs, multifactorial interventions including staff education reduced the number of fallers and recurrent fallers [5, 17, 29], but the effectiveness of staff education as a single intervention was inconsistent [5, 17, 29]. A recent scoping review synthesised the evidence on staff educational fall prevention interventions across multiple study types, and described the education programme contents and characteristics [30]. It concluded that few studies were underpinned by comprehensive educational designs and that the educational programme standard was low. One key part of designing an effective educational intervention for interdisciplinary staff is identifying potential deficiencies in staff knowledge and skills (an objective “education need”) [31], such that one discipline or staff grade may need more time or different content than another in a topic. Just as important is the desire to learn more (regardless of baseline knowledge), i.e. the learner’s subjective learning need. The latter is defined as a knowledge gap between what a learner wants and needs to know, and already knows [32]. The latter has complex influences, including attitudes (perceived importance of the topic), confidence, and prior experience, and aligns with a social constructivist approach to education, where-in the attitude, motivation and experience of the whole learning group will influence the individual learner’s interaction with and application of an education offering. Thus, it is crucial to identify attitudes, confidence, and interest in facilitating fall prevention [30, 33]. In one descriptive study, LTCF staff lacked knowledge in assessing and treating intrinsic fall risk factors although 81.6% (120/147) considered falls a serious issue in LTCFs in general, and 39 of them believed that their facility faced a serious falls problem [34]. On the face of it, such a group would be very motivated to learn, but would need significant support in their learning, given the low knowledge level in the peer group. Another study reported that LTCF staff lacked knowledge and awareness of fall prevention interventions, had a low level of capability in terms of assessing residents with a low or moderate risk of falls, and lacked sufficient staffing levels [33]. It is also recommended that the self-efficacy of nursing staff should be assessed [35], where they may be motivated to reduce the falls rate in LTCFs, but lack the knowledge and training to carry out fall prevention activities. from passive acceptance of falls, to active engagement in falls prevention.

### Aims and objectives

The aim of the present study was to explore LTCF staff knowledge, attitudes and confidence about fall prevention among residents, to inform a future tailored educational intervention, and in particular:

- To examine the fall prevention knowledge of LTCF staff and identify knowledge gaps based on their job role (i.e. their education needs).
- To explore whether or not staff knowledge differs according to demographic factors (i.e., role, experience, seniority or prior education/training), so that education needs could be anticipated from staff characteristics prior to any pre-learning tests (to facilitate initial content/delivery/assessment development).
- To explore staff attitudes towards falls in LTCFs and their confidence and motivation to conduct fall prevention activities (which would influence learning needs and hence the delivery of the education).
- To explore prior education training taken by LTCF staff and their preferences for future education training (to tailor content and delivery to user preferences).

## Methods

The current study is one part of an overall mixed-methods sequential explanatory design study, conducted in LTCFs, which seeks to explore LTCF staff’s knowledge and practice regarding fall prevention and the perceived barriers to improving fall prevention, to tailor future staff education and skills training. The reporting of the current study used the Strengthening the Reporting of Observational Studies in Epidemiology (STROBE) guidelines [36, 37].

### Design

This was a cross-sectional, descriptive study using a survey designed to identify the knowledge gap, attitudes and confidence levels related to fall prevention, in addition to identifying their training needs in the future.

### Setting

The setting comprised the community healthcare region within which our university is based, and for whose staff the university commonly provides education and training. This healthcare region is made up of the counties of Cork and Kerry, in southwest Ireland. Cork is geographically larger and more densely populated, with a mixed urban-rural population, while Kerry has a much smaller, older and mainly rural population. These counties have shared funding, governance and delivery of all community-based healthcare, which includes residential care. All LTCFs for older people (n = 71), comprising 65 sites in Cork and six in Kerry, were eligible (total bed number approximately 3660, from a total of 31,900 beds nationally) [38].

### Selection of sites

A sampling framework was used to seek variation with regard to the provider type, across private providers (for-profit, the most common provider type in Ireland), voluntary (not-for-profit, state-funded, with a charitable ethos +/- top-up funding from donations, which is a rare type) and public (not-for-profit, state-funded and state-provided). It also sought variation in the size of the facilities, divided into more than 50 beds (the most common) or fewer than 50 beds, and in the location (urban/rural; Cork/Kerry). Sites were selected within each sampling group using the random sample function in Microsoft Excel. A 20% LTCF sample (n = 14) was chosen, including six public sites (five in Cork), six private sites (five in Cork) and two voluntary sites (both in Cork, as Kerry does not have any voluntary sites). These also included nine large sites and five small sites, and there was equal distribution in terms of urban/rural locations.

### Recruitment of sites and participants

A researcher contacted 14 randomly selected sites and shared study information by phone and email. An invitation letter with a link to the survey and promotional study posters was sent to each site that agreed to take part.

Within each LTCF, the staff included the Director of Nursing (DON), ward nurse managers, staff-grade nurses, healthcare assistants, physiotherapists, occupational therapists, other health and social care professionals, and visiting General Practitioners (GPs) or medical officers. Participants had to be working full- or part-time at the site for at least three months to be eligible. All staff that met the inclusion criteria at a site were invited to participate.

### The survey instrument

Our survey instrument was informed by the specific research aim, and guided by socio-constructivism learning theory (that knowledge strongly relates to the context and culture in which it is formed and used) and by behaviour change theory (that attitudes, confidence, motivation, etc. are also key to positive changes in staff behaviour). Thus, our survey aimed to explore staff knowledge deficits, but also attitudes, motivation and confidence. The survey consisted of **38** questions, which merged two existing evidence-based surveys, as follows:

- Fall Knowledge Test: Form 2E from the Agency for Healthcare Research and Quality (AHRQ; https://www.ahrq.gov/patient-safety/settings/hospital/fall-prevention/toolkit/fall-knowledge-test.html) [39]was adopted without change. This had been specifically developed to determine staff knowledge of fall prevention in LTCFs based on a national fall prevention guideline [40]. All 13 questions were included in our survey.
- A previous survey used the Capability, Opportunity, and Motivation to Undertake a Health Behaviour Change (COM-B) framework to evaluate LTCF staff knowledge, confidence and attitudes regarding fall prevention interventions [33, 34]. This survey had been validated by five staff members in LTCFs using content validity testing, and English literacy requirements were checked. We used 22 questions from the COM-B survey (out of 36 questions) with minor wording modifications only to fit the aim of the study (see Supplementary File 1). We excluded knowledge questions (as already assessed using Form 2E questions) and specific questions on ‘assessing reminder systems’, which was specific to the genesis project for that survey, but not to our context.
- We also added 3 new questions, one of which was related to demographics (job role) and the other two were open-ended questions (staff suggestions and comments for fall prevention activities (see Supplementary File 1).

The survey contained a mixture of **closed-ended (n = 31) and open-ended (n = 7)**; the time for completion was approximately 10–15 min.

The survey content included the following:

- Demographic data (n = 7): age, gender, educational level, job role, years of experience working with older people and of working in this LTCF, shift work pattern.
- Knowledge-related questions (n = 13): multiple-choice questions, with more than one correct answer, for a total possible score of 33 points.
- Attitude and confidence items (n = 7): self-rated using a five-point Likert scale.
- Previous training and future educational preferences (n = 4): closed-ended questions, with one open-ended question on learning methods in any previous fall prevention training.
- Open-ended questions: staff solutions for fall prevention activities (n = 2); current practice regarding fall prevention (n = 2); two other open-ended questions asked for suggestions for fall prevention activities in LTCFs, which will not be represented in this paper (as data was co-analysed with other qualitative data).

The online survey was developed using Microsoft Forms and two nurses and one physiotherapist pilot-tested it for clarity of instructions, and question structure and wording. Based on their feedback, minor wording changes for clarity were made. They also timed how long it took to complete the survey to inform the participant information text.

A paper version was also prepared, as senior site staff indicated that this would facilitate staff preference and overcome potential computer/internet issues.

### Data collection

The self-administrated survey was circulated by a site champion, an employee chosen by the DON to collaborate with the primary researcher (NA). For the online survey, the champions received invitation emails, with details about the study, and a survey link to be circulated via the site’s social media or staff email addresses. We also distributed 540 paper surveys by post, with a request to return the completed questionnaires within four weeks, using the enclosed postage-paid envelopes (2–3 such envelopes allowed for waves of response). Champions received two reminder emails to encourage and remind participants, after one and three weeks. The GPs were recruited with assistance of the site DONs, as being the site’s identified GP or medical officer, and the online link and/or printed survey with the invitation letter were sent to them. The overall recruitment period for this study was four months, from April to August 2022. Online and paper-based replies were merged in a single database.

### Data analysis

#### 1: quantitative data

All quantitative data were analysed using IBM SPSS (Statistical Package for the Social Sciences) for Windows, software version 28, and were summarised using descriptive statistics. Categorical data were reported as percentages and frequencies, while numerical data were reported as median values with Interquartile Range (Q1, Q3), as data were not normally distributed.

### Analysis of fall prevention knowledge

The AHRQ confirmed that the total possible score was **33**, with 1 point for any correct answers and 0 points for incorrect or unanswered questions. In determining knowledge gaps, for a given question, respondents who gave any incorrect answers were taken as having insufficient knowledge of that topic, regardless of the exact number of correct/incorrect items within the question. We excluded three cases from analysis where partially completed knowledge tests resulted in outlying scores. The overall level of fall prevention knowledge was classified as “inadequate” for any score of 16 or less (i.e., less than 50% of the total possible marks), and “moderate knowledge” if 17 to 25 (50–75% score) or “adequate knowledge” if 26 or more (more than 75% score).

We performed a cross-tabulation of the items most often answered incorrectly, comparing to the staff members’ roles (e.g., healthcare assistants would be expected to be less familiar with comprehensive assessments or medication). We used non-parametric Mann-Whitney U and ANOVA Kruskal-Wallis tests to analyse the differences between the overall scores and demographic variables. For gender and age categorical groups, we excluded those that selected “prefer not to say”. For prior education, we combined the three categories of “master’s degree”, “postgraduate certificate and “postgraduate diploma” into one new category: “postgraduate level” as numbers were small and the education level for these is similar, noting also that this postgraduate education may not have included fall prevention. We also merged the categories of DON and senior nurse, as we expected that they would have similar levels of knowledge. The data for GPs, health and social care professionals (HSCPs), and administrators, are presented but excluded from comparative analysis due to very small numbers. Within ‘years of experience working with older people in healthcare’, we combined “less than one year” and “1–2 years” as a new category of “two years and less”, due to small sample size. All tests were conducted using a 95% confidence level, and the significance level was set at .05.

A univariate linear regression model was developed to examine which parametric variables most explained the variation in total knowledge scores. The explanatory variables included gender, years of experience working with older people, level of prior education, job role and previous training in fall prevention. We excluded age and years of experience working in LTCFs variables from the model, because age would overly overlap with years of experience, while specific experience in that particular LTCF may not accurately reflect clinical professional experience in fall prevention in other LTCFs or other settings.

A stepwise, backwards, regression model examined the strength of influence of parameters on knowledge. Firstly, we explored the association between explanatory variables using the Pearson chi-square test to identify any multicollinearity, excluding any variable that was highly associated with other variables. To confirm the assumption of normality, the residuals were analysed; these were not normally distributed, being negatively skewed on scatter plots [41]. A log 10 reflective transformation was made as per the following formula: *Reflection = (X Max + 1 – Xi)*, where *X Max* is the highest value of the respondents’ knowledge score and *Xi* is each total knowledge score [42, 43]. The analysis was repeated using backward elimination to identify the most insignificant variables via three, sequential steps, leading to the variable that had the most significant impact on staff fall knowledge.

### Staff confidence and attitude analysis

We used non-parametric Mann-Whitney U and ANOVA Kruskal-Wallis tests to analyse the differences in staff attitude and confidence levels according to demographic variables.

### Sensitivity analysis

The facility response rate varied across sites, so we divided all of the sites into two groups based on the median response rate (high response rate group: 30% or more; low response group: lower than 30%) and compared data from both groups, to explore any differences due to response rate, and hence possible responder bias.

### 2: qualitative data

Qualitative data from open-ended questions were analysed using content analysis [44, 45] using NVivo Version 2021, using both inductive and deductive approaches. An initially inductive approach grouped specific observations into general statements/themes, based on the meaning of the words in the text. For this, open-ended questions were coded and categorised independently by two researchers; they compared and discussed their findings initially, and the results were discussed with the research team to encourage reflection. Then, using a deductive method, similar subcategories were categorised using fall prevention domains from the fall literature. Frequency responses were also recorded. To obtain a better understanding of the differences between LTCF staff in terms of their approaches to preventing falls and fall-related injuries, and their current practice, we conducted a cross-tabulation of the general themes with job role. This aimed to inform the development of future, role-targeted education.

### Ethics

Ethical approval was obtained from the Social Research Ethics Committee (SREC) at University College Cork (UCC). The survey was fully anonymous and did not ask for any personal data. Participation was voluntary; participants were provided with study information at the beginning of the survey and if they were happy to proceed, they were asked to tick a consent box. Hard copy data was stored in locked cabinets accessible only to the researchers. Online survey data was stored in password-protected university hard drives.

## Results

### Participants’ characteristics

Overall, 13 out of 14 invited LTCFs agreed to participate in this study, representing 93% of the invited sites. This final sample was 18% of all sites in the region, and included 28% of older person residential bed numbers in the region. In total, approximately 1,039 staff were working across these sites. Within these, it is not known how many staff were ineligible based on working at the site for less than 3 months; thus, the overall survey response rate was at least 15% (n = 155), ranging from over 1% to over 55% per site. Assuming that the proportion working less than 3 months in LTCFs was similar to the proportion working 3–6 months among the respondents (i.e., 13.5%), the eligible pool was 898 staff, and the response rate was thus 17%. Participant demographic data are described in Table 1. The majority were female (n = 122, 78.7%), while most staff were aged 30–59 years (30–39 years: 27.7% of staff; 40–49 years: 22.6% of staff; 50–59 years: 23.2% of staff).

**Table 1:** Participant demographics

Demographics	N(155)	100 (%)
Gender		
Male Female Prefer not to say	31	20
	122	78.7
	2	1.3
Age		
18–29 years 30–39 years 40–49 years 50–59 years 60–65 years Prefer not to say Missing	28	18.1
	43	27.7
	35	22.6
	36	23.2
	9	5.8
	3	1.9
	1	6
Education level		
FETAC level Bachelor Post-graduate Certificate Post-graduate Diploma Master Other Missing	40	25.8
	67	44.4
	6	3.9
	21	13.5
	15	9.7
	2	1.3
	4	2.6
Job Role		
Senior Nurse/CNM Nurse Healthcare assistants General practitioner HSCP Other Missing	32	20.9
	51	32.9
	55	35.5
	8	5.2
	2	1.3
	5	3.2
	2	1.3
Experience as a (paid) carer for older people		
< a year 1–2 Year 3–5 year 6–10 year > 11 Year Missing	25	16.1
	20	12.9
	24	15.5
	18	11.6
	66	42.6
	2	1.3
Experience in their long-term care facility		
3–6 Months 7–12 Months 1–2 Years 3–5 years 6–10 years >11 Years	21	13.5
	14	9
	34	21.9
	22	14.2
	21	13.5
	43	27.7
Works shifts		
One shift ^a^ Two shifts ^b^ Three shifts ^c^ Four shifts ^d^ Five shifts ^e^	72	46.5
	50	32.3
	14	9
	5	3.2
	5	3.2

*Note*: FETAC: Further Education and Training Awards Council; CNM: Certified Nurse Midwife; HSCP: Health social and care professional

^a^ Reflects the total number of respondents who work one shift as follows: Morning (n = 7), Full Day (12 h) (n = 53), Night (12 h) (n = 8), and Twilight hours (n = 4)

^b^ Reflects the total number of respondents who work two shifts as follows: Morning/ Afternoon (n = 16), Morning/ Full day (n = 1), and Full day/ Night (n = 23)

^C^ Reflects the total number of respondents who work three shifts as follows: Morning/ Afternoon/ Full day (n = 7), and Morning/ Full day/ Night (n = 7)

^d^ Reflects the total number of respondents who work four shifts as follows: Morning/ Afternoon/ Full day/ Night (n = 3), and Morning/ Afternoon/ Full day/ Twilight (n = 2)

^e^ Reflects the total number of respondents who work four shifts (i.e., Morning/ Afternoon/ Full day/ Night/ Twilight)

As expected, the most frequent respondent discipline as nursing staff, with 51 (32.9%) nurses and 32 (20.9%) senior nurses, including seven DONs. The other large group were HCAs (n = 55; 35.5%). Of the remainder, eight were GPs, two were physiotherapists, and five were “other” disciplines including three administrators and two maintenance staff. Sixty-six (42.6%) had more than 11 years of experience working with older people in healthcare. However, the majority of respondents had worked in their current LTCF between three months and two years (n = 69), while 43 had worked there for 11 years or more.

The most common education level was a bachelor degree (i.e., European Qualification Framework level 6; n = 67, 44.4%; predominantly nursing staff), followed by further education awards at European Qualification Framework level 4/5 (n = 40, 25.8%; predominantly healthcare assistants). Seventy-two (46.5%) worked a limited shift (e.g., morning and afternoon, or morning only, or twilight hours only); the most common shift was a full day (12 h), while eight staff worked only at night.

### Staff knowledge of falls

Excluding the three non-completers (n = 152), the median score in the falls knowledge test was 26 [IQR 24 to 30, range: 8–33]; with 60% (n = 89) deemed to have adequate knowledge (score range: 26–33),34% (n = 52) having moderate knowledge, and 7% (n = 11) having inadequate knowledge (score range: 16 or less). Of these 11 staff, six were nurses, four were HCAs, and one was an administrator. Overall, 78.1% (n = 25) of senior nurses and 5 of the 8 GPs had an adequate level of knowledge (see Table 2).

**Table 2:** Fall knowledge level, according to staff job roles

LTCF staff job role	Knowledge level						Total
	Inadequate Knowledge		Moderate knowledge		Adequate knowledge		
	N	(%)	N	(%)	N	(%)	
Senior nurse	0	0.0	7	21.9	25	78.1	32
Nurse	6	11.8	20	39.2	25	49.0	51
HCA	4	7.7	19	36.5	29	55.8	52
GP	0	0.0	3	37.5	5	62.5	8
Physiotherapist	0	0.0	0	0.0	2	100	2
Others	1	20.0	2	40.0	2	40.0	5
Total	11	7.3	51	34.0	88	58.7	150

*Note*: HCA: Healthcare assistant; GP: General practitioner

### Areas where fall prevention knowledge was poor

Table 3 displays the percentages of correct answers for each of the 13 questions in the fall knowledge test. There was poor knowledge of some interventions targeting individual modifiable falls risk factors such as medication review, regular toileting, avoiding antipsychotic medication, using mobility aids, and exercise programmes. The test item on keeping confused residents nearer the nursing station was also poorly answered, noting that this item contained an implied “double negative” wording (see item wording in Table 3). Similarly, the item on using a patient identifier to highlight those at high risk of falls was stated as ‘a patient identifier, e.g., an identification bracelet’ which could simply refer to a patient’s ID bracelet being used to identify residents by name (which would be unlikely to reduce falls), rather than a falls-identification system such as a specially coloured bracelet. Other poorly answered questions related to the discipline with responsibility for fall prevention, where many erroneously agreed with the stated item that only nurses are responsible, and the value of performing a post-fall analysis.

**Table 3:** The percentage of correctly answered questions for each point on the staff knowledge test

Fall Knowledge questions	The main items of questions	Total scored correctly (152)	(%)
Q1: Which of the following correct statement	Falls have multifactorial aetiology, so fall prevention programs should comprise multifaceted interventions	139	91.4
	Regular review of medication can help to prevent patient falls ^a^	103	67.8
	The risk of falling will be lessened when resident toileting needs are met ^a^	93	61.2
	The use of antipsychotic medications is associated with an increased risk of falls in older adults^b^	91	59.9
Q2: A multifaceted intervention program should include	Individually-tailored fall prevention strategies	133	87.7
	Education for residents/family and healthcare workers	127	83.6
	Environmental safety	130	85.5
	Safe patient handling	128	84.2
Q3: The risk factors for falls in the nursing home include all of the following except	Antibiotic usage	111	73
Q4: Which of the following correct statement	The cause of a fall is often an interaction between resident risk, the environment, and patient risk behaviour	112	73.7
	An increase in hazardous environments increases the risk of falls	113	74.3
	The use of a patient identifier (e.g., an identification bracelet) helps to highlight to staff those residents at risk for falls ^b^	56	36.8
	A fall risk assessment should include a review of the history of falls, mobility problems, medications, mental status, continence, and other resident risks	142	93.4
Q5: Resident with impaired mobility should be	Encouraged to mobilize with assistance	130	85.5
	Assisted with transfers	115	75.7
	Referred for an exercise program or prescription of walking aids as appropriate	132	86.8
Q6: The management of the acutely confused resident should include all the following except	Moving residents away from the nursing station ^b^	72	47.4
Q7: Which of the following false statement	Fall prevention efforts are solely the nurses’ responsibility ^a^	95	62.5
Q8: In long-term care settings, intervention programs should include	Staff education on fall precautions	109	71.7
	The Provision and maintenance of mobility aids ^a^	99	65.1
	Post-fall analysis and problem-solving strategy ^a^	104	68.4
Q9: Which of the following false statement	Environmental assessment is not important in the nursing home as it is all standardized	134	88.2
Q10: Risk factors for falls include	Parkinson’s disease	140	91.4
	Incontinence	125	82.2
	Previous history of falls	144	94.7
	Delirium	139	89.70
Q11: Exercise programs for ambulatory older adults should Include	be ongoing ^a^	103	67.8
	individualized strength and balance training	129	84.90
Q12: Which of the following false statement	Education should not only be given at the start of the fall prevention program	138	90.8
Q13: Which of the following is recommended to improve resident safety	Locking wheeled furniture when it is stationary	134	88.2
	Having nonslip flooring	140	92.1
	Placing frequently used items (including call bell, telephone, and remote control) within reach of the resident	141	92.8
	Rounding hourly to address resident needs	122	80.3

^a^ reflects all items answered correctly by less than 70% of respondents

^b^ reflects all items answered correctly by less than 60% of respondents

### Staff knowledge gaps across different disciplines and roles

Table 4 shows the performance, by job role, for the nine items that staff knew the least about. Overall, nurses in non-senior roles performed worst in these items, and HCAs performed worst in two topics (i.e., moving confused residents near a nurse station and staff responsibility regarding fall prevention efforts). Of note, all job roles had the lowest test performance in the item about keeping the confused residents close to a nurse station and using resident identification to highlight who was at high risk. As expected, GPs performed best for the two questions relating to medications, but overall, the physiotherapists had very good levels of falls knowledge, followed by GPs and “others”, then senior nurses.

**Table 4:** Staff who incorrectly answered the “least well answered” items in the fall knowledge test, categorised according to job role

The knowledge items most often answered incorrectly	Incorrect responses: N () and % of total in that staff category	Discipline / Role (total N for each staff category)					
		Senior nurses (32)	Nurses (51)	HCAs (55)	GPs (8)	HSCPs (2)	Others (5)
Regular review of medication can help to prevent patient falls	N (48)	7	21	17	0	0	3
	%	21.9	41.2 ^b^	32.7	0.0	0.0	60.0 ^b^
The risk of falling will be lessened when resident toileting needs are met	N (58)	8	28	17	2	0	3
	%	25.0	54.9 ^b^	32.7	25.0	0.0	60.0 ^b^
The use of antipsychotic medications is associated with an increased risk of falls in older adults	N (59)	7	25	22	1	0	4
	%	21.9	49.0 ^b^	42.3 ^b^	12.5	0.0	80.0 ^b^
The use of a patient identifier (e.g., identification bracelet) helps to highlight to staff those residents at risk for falls. ^a^	N (95)	19	34	31	6	1	4
	%	59.4	66.7	59.6	75.0 ^b^	50.0	80.0 ^b^
Moving confused residents away from the nursing station. ^a^	N (78)	14	20	34	4	1	5
	%	43.8	39.2	65.4 ^b^	50.0	50.0	100 ^b^
In long-term care settings, intervention programs should include the provision and maintenance of mobility aids	N (52)	6	25	17	3	0	1
	%	18.8	49.0 ^b^	32.7	37.5 ^b^	0.0	20.0
In long-term care settings, intervention programs should include Post fall analysis and problem-solving strategy	N (47)	5	25	15	2	0	0
	%	15.6	49.0 ^b^	28.8	25.0	0.0	0.0
Exercise programs for ambulatory older adults should be ongoing	N (49)	5	24	17	2	0	1
	%	15.6	47.1 ^b^	32.7	25.0	0.0	20.0
Fall prevention efforts are solely the nurses’ responsibility	N (56)	12	18	22	2	0	2
	%	37.5	35.3	42.3^b^	25.0	0.0	40.0

^a^ reflects all items answered poorly across all disciplines and job roles

^b^ indicates the job role(s) answering worst for that item

### Factors influencing fall prevention knowledge

Seven independent variables were examined for influence on fall knowledge total scores; there was a statistically significant difference in performance according to the respondent’s job role (H = 9.153, p = .010) and years of experience working with older people (H = 19.733, p = .001) (see Supplementary File 2). Senior nurses had significantly more knowledge about fall prevention (median 29) than nurses (median 26; p = .015) and HCAs (median 27; p = .027) (see Table 5), while no significant difference existed between nurses and HCAs. The median score for GPs was 27.5.

**Table 5:** Pairwise comparison of the fall knowledge test according to job role and years of experience

Pairwise Comparisons of the job role	Test Statistic	Std. Error	Std. Test Statistic	Sig.	Adj. Sig. ^a^
Nurses versus HCAs	-1.838	7.683	− 0.239	0.811	1.000
Nurses versus Senior nurses	24.725	8.792	2.812	0.005	0.015*
HCAs versus Senior nurses	22.887	8.760	2.613	0.009	0.027*
**Pairwise Comparisons of years of experiences working with older people**	**Test Statistic**	**Std. Error**	**Std. Test Statistic**	**Sig.**	**Adj. Sig.** ^**a**^
less than or equal to 2 years versus 6–10 years	-28.080	12.119	-2.317	0.020	0.123
less than or equal to 2 years versus 3–5 years	-29.286	11.145	-2.628	0.009	0.052
less than or equal to 2 years versus 11 years and more	-36.853	8.456	-4.358	< 0.001	0.000**
6–10 years versus 3–5 years	1.207	13.630	0.089	0.929	1.000
6–10 years versus 11 years or more	-8.773	11.536	− 0.760	0.447	1.000
3–5 years versus 11 years or more	-7.567	10.508	− 0.720	0.471	1.000

*Note*: Each row tests the null hypothesis that the Sample 1 and Sample 2 distributions are the same (e.g. Nurses versus HCAs). Asymptotic significances (sig; 2-sided tests) are displayed. The significance level is 0.050. Std = standard

a. Significance values adjusted using the Bonferroni correction for multiple tests

P*>0.05, p**>0.01

The median fall knowledge score increased with more years of clinical experience (see Supplementary File 2). Respondents with 11 years or more clinical work experience with older persons had better fall prevention knowledge than those with the least amount of experience, namely two years or less (p = .000) (see Table 5). In addition, there was a clear trend towards better scores with higher educational levels; whereas the increase in scores with higher age and more years of work experience in the particular LTCF appeared to plateau after initial increases (see Supplementary File 2).

### Independent influences on fall prevention knowledge

When exploring the association between explanatory variables (i.e., gender, educational level, years of clinical experience, job role and previous fall training), we identified that the respondent’s job role was highly associated with their educational level and with years of experience working with older people. Gender was also associated with job role, although not to the same degree. Thus, job role was excluded from the model, and the remaining explanatory variables were included.

Following a univariate linear regression model fitting, and a log transformation of the residuals see methods), the analysis was repeated. Stepwise backward elimination of the least significant variables, started by excluding gender, then previous fall training, and then educational level. In the final model, years of clinical experience working with older people significantly influenced fall knowledge scores (R^2^ 74.623, F = 6.644 and p < .001). In post hoc analysis correcting for multiple comparisons, experience of 11 years or more had a statistically significant impact on fall knowledge scores compared to experience of two years or less. (See Supplementary File 3 for more details on these statistics.)

### Staff attitudes toward falls in LTCFs

**Fig. 1:**
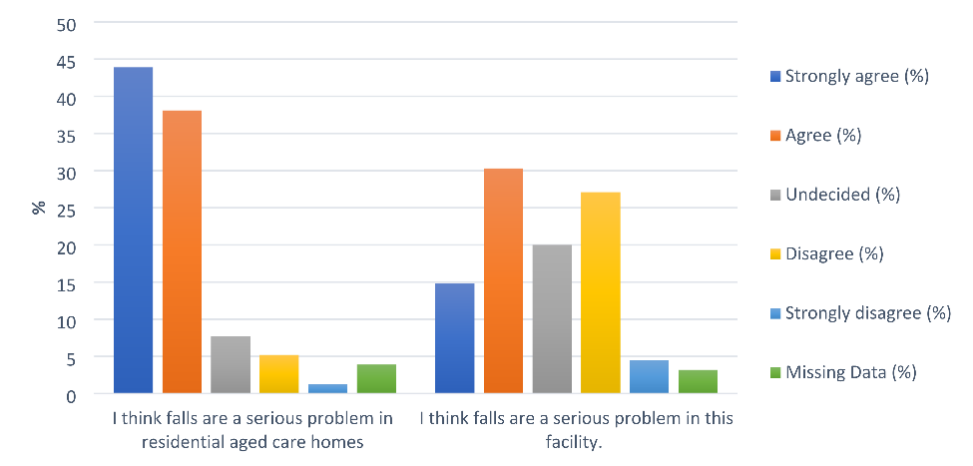
Staff attitudes related to viewing falls as a serious problem in LTCFs

Among the 149 staff members who answered this question, 43.9% (n = 68) strongly agreed and 38.1% (n = 59) agreed that falls were a serious problem in LTCFs in general (total = 85%), and only 6.5% disagreed. However, only 45% of respondents (n = 70) agreed or strongly agreed that falls were a serious problem in their own facility, and approximately 32% [49] disagreed with this statement (see Fig. 1). For the options given for the rate of falls annually, 43.2% (67) correctly chose that 50% of residents in LTCFs fell every year, while 36.1% chose the option of ‘20%’ and 5.8% chose ‘10%’ and 15.8% either chose the ‘unsure’ option or did not answer.

Staff attitudes differed statistically significantly between those who had previously completed fall training and those who had not (p = .001) with regard to falls being a serious issue in their own LTCF, where almost 60% (46/77) who had training agreed or strongly agreed with this statement, compared to 28% (15/50) of those who had no prior training.

### Staff confidence for conducting fall prevention activities

**Fig. 2:**
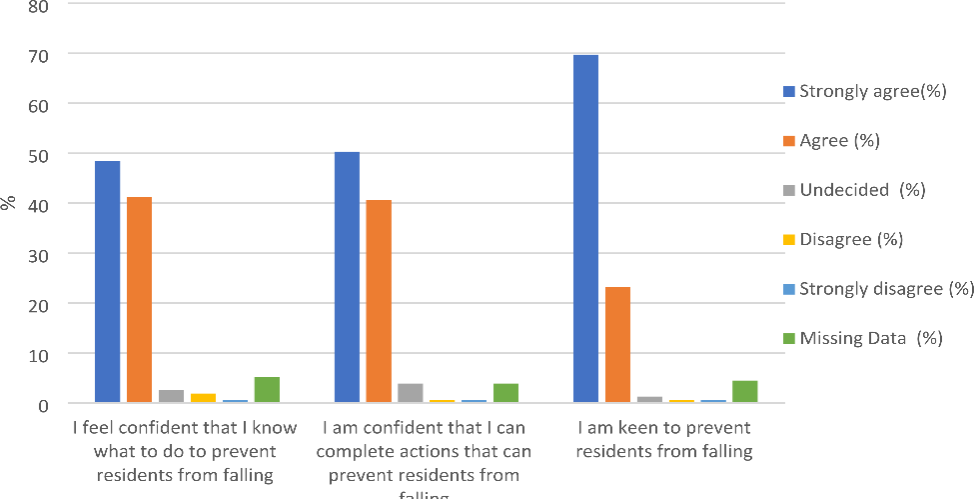
Staff confidence levels and motivation to conduct fall prevention activities

Almost 90% of respondents reported a high degree of confidence (i.e., answering “strongly agree or agree”) in their skills to help residents avoid falls and also to carry out all prevention actions. The few dissenters included nurses and HCAs (see Fig. 2).

Confidence in carrying out fall prevention activities increased as clinical years of experience working with older people increased (p = .010; see Supplementary File 4). Respondents with 6–10 years of clinical experience working with older people had higher levels of confidence than those with three to five years of clinical experience (p = .025), but lower levels of confidence levels than those with 11 + years of experience.

### Prior fall prevention education and future training preferences

Approximately 50% of the respondents (n = 78) had completed fall education and training in the previous five year, while 15.5% were unsure of this. Senior nurses, including DONs, most often recalled such training, at almost 60% (19/32), followed by HCAs and nurses, at 52.72% (26/51) and 50.98% (29/55), respectively. In total, none of the eight GPs had received any recent fall education and training. Nearly 50% of respondents thought they had enough fall prevention training, while 25% indicated that they needed more training (see Supplementary File 5).

There were large variations reported in the respondents’ prior fall prevention training, in terms of the duration of the training, topics covered, location, educational material and speakers (n = 70 replies; Supplementary File 6). Where reported, most had attended training sessions that lasted for several hours and these were conducted mostly during work hours. Face-to-face education was almost twice as common as online, and was mainly provided on-site. Lectures and slide presentations were often utilised. Typically, a combination of online and paper educational resources was provided, and education focussed most often on fall prevention education, fall risk assessment and manual handling techniques. Where specified, education was most often delivered by a HSCP or nurse.

Considering future education and training, of 113 respondents, 81 chose in-service training in their facilities as their sole learning preference while 30 chose only e-learning, and 2 chose only watching DVDs. Within those who selected two preferred modes of delivery (n = 19), in-service training combined with e-learning was the most popular (n = 13), with the remainder choosing a combination involving DVDs and either e-learning or in service (2 and 4 respondents, respectively) (see Supplementary File 5).

In the sensitivity analysis to detect potential differences between respondents from sites with higher and lower response rates, there was no significant difference between the two groups in terms of median score in the Falls Knowledge Test (p = .493). However, there were statistically significant differences between respondents in terms of confidence in the following: (i) having skills to help residents avoid falls, with more confidence in respondents from sites with low response rates compared to those with high (p = .005); (ii) carrying out fall prevention activities, with again more confidence in respondents from sites with low response rates (p = .019). Furthermore, there was a trend towards a higher frequency of concern with regard to falls being a serious problem in LTCFs in general (p = .055) and for falls being a serious issue in their own LTCF (p = .103), and for motivation when conducting fall prevention activities (p = .263), in the respondents from sites with low response rates. To summarise, respondents in low-response sites were not more or less knowledgeable, but were more confident, concerned and motivated to prevent falls, indicating a possible response bias in low-response sites.

## Discussion

Assessing and treating residents at risk of falling or recurrent falls is regarded as a vital aspect of the work of healthcare professionals. A lack of staff knowledge of fall risk factors can be viewed as a risk factor for falls, as healthcare professionals’ knowledge and awareness of falls and fall-related risk factors are predicted to have a major influence on preventing falls among residents [22]. Similarly, LTCF staff’s attitudes are considered essential for successfully translating evidence-based recommendations into practice [33]. This study thus aimed to investigate staff knowledge and attitudes regarding fall prevention in LTCFs, and has provided new insights into the knowledge gap among LTCF staff for fall prevention, and among certain disciplines and job roles.

Our findings demonstrate that LTCF staff had an overall adequate level of fall knowledge, as measured across many learning and clinical domains related to fall prevention strategies, most of which are included in best practice guidelines on preventing falls [1, 8, 22, 46]. Our sample scored better than hospital nursing staff, using a similar fall-knowledge test, where almost 60% had an inadequate level of knowledge (scores of 16 or less out of 33 points) [47]. Additionally, our findings indicate that LTCF staff members’ increasing years of clinical experience caring for older people increased their knowledge of falls; i.e., that practical clinical experience can increase knowledge [48]. Previous studies examining staff knowledge of fall prevention among older people in other settings also found that staff knowledge was affected by practical experience [21, 49].

This study shows that LTCF staff knowledge of fall prevention had some gaps, such as the value of regular medication reviews to prevent falls, and not knowing that antipsychotic medication can increased residents’ risk of falls. A previous qualitative study had similarly found that frontline nursing home staff needed more training on medication-related falls [50], while another showed that hospital staff’s lowest levels of falls knowledge related to disease and medication-related falls [49]. It is known that nursing staff in various settings lack knowledge of the side effects of antipsychotic medication use [51]. Psychotropic medications are linked to an increased risk of falls, with the relative risk ranging between 1.5 and 1.7 [6, 52]. Residents in LTCFs are frail with a higher incidence of all types of falls, particularly those linked to medication, compared to other groups [52]; thus, all LTCF staff should be supporting medication reviews and the appropriate use of antipsychotic drugs, which can be promoted within LTCF staff fall education.

Ongoing exercise programmes for ambulatory residents and the correct provision of mobility aids were identified as areas of least knowledge among LTCF staff. This reflects a previous qualitative study of LTCF staff, who had a lack of knowledge about the importance of exercise regimens and equipment safety to prevent falls and maintain resident independence [53]. Exercise improves muscular strength, balance and coordination and is vital for bone health. It is recommended that LTC residents engage in an individual, multimodal exercise program at least twice per week for 35 to 45 min per session [54]. This emphasises the necessity of further education focusing on these specific topics, which can then be enacted via multidisciplinary approaches.

Interestingly, our findings highlight how LTCF staff were least knowledgeable about the value of post-fall analysis and fall prevention problem-solving strategies. Critically examining each fall incident, circumstance and the possible root cause is recommended to identify particular risk factors in an individual resident and to give an in-depth understanding of how and why residents fall [22]. Because the aetiology of falls in LTCFs is typically multi-faceted, effective fall prevention techniques and care plans can be developed and put into place using these recognised risk factors [55]. Problem-solving techniques incorporating multi-disciplinary teams can prevent falls and fall-related injuries, as demonstrated in multi-factorial interventions [56]. This approach is crucial to clinical practice for preventing falls and lowering hospitalisations [6]. Additionally, meeting residents’ toileting needs is one of the strategies identified to lower the risk of falls. However, this study shows that LTCF staff knowledge in this area was insufficient. If it is determined that urinary urgency or incontinence are prompting hazardous transfer or ambulation, a toileting plan could lessen this behaviour [57]. Timed voiding is a defined time-interval toileting assistance programme that can support residents to maintain their continence, and it may also help to reduce their risk of falls [58, 59]. Thus, toileting issues should be a domain within a fall education programme.

Fall prevention is a team effort that often includes the entire LTCF, where all of their input is crucial. Our results show that senior nurses have better knowledge about falls than others, related to their duration of clinical experience, and reflecting their greater responsibility for environmental management, human resources, health and safety, etc. [60]. However, our study found that some nurses and HCAs had significant gaps in knowledge, consistent with earlier findings [50, 53]. Implementing fall preventive programmes is primarily attributed to nurses in LTCFs, particularly in the assessment of falls [61]. However, healthcare assistants assist residents with the activities of daily living (ADLs), such as bathing, dressing, toileting, ambulation and feeding; they are in charge of providing direct care and are required to proactively use fall prevention strategies with residents [61]. It has been estimated that they spend 45.4% of an eight-hour shift on direct care, compared to allied health professionals, such as physiotherapists, who spend an average of 2.3% of an eight-hour shift on direct care in this setting [62, 63]. They must thus have the necessary training to recognise risk factors and use preventative measures while they are continuously caring for residents, and further, tailored, fall prevention education is indicated.

While fall knowledge is important, other considerations are also important. One strategy is to increase the monitoring of residents, such as keeping confused residents near the nurse station, particularly those who have cognitive impairment or delirium [40, 63]. Our study found an apparent poor knowledge of the value of this strategy, but this may have related to the particular “double-negative” wording of this statement, or to the practical challenge of moving about residents’ rooms, particularly if many are at increased risk of falls. Another strategy is to use an identifying system for residents who are at high risk of falling, such as coloured identification bracelets, to increase staff awareness of a particular resident’s fall risk [40]. Again here, our sample answered this question poorly, but this may also have related to the wording, wherein it may have appeared to refer to resident identification bracelets, rather than specific falls-alert bracelets. Finally, due to the complexity of fall prevention, all LTCF staff are responsible for overcoming fall-related health issues in LTCFs, but many of our sample considered this to be a nursing role only [40]. Thus, future staff education should quickly assess the learners’ knowledge of the value of close monitoring and falls-alert systems, and provide learning if required, and the need for a whole-team approach to falls needs to be promoted.

Residents in LTCFs have vulnerable characteristics, which require staff to be knowledgeable and to implement high levels of preventive activities to maintain patient safety. Changing staff attitudes and behaviours about falls is crucial to incorporating knowledge into daily clinical practice [64]. Fall prevention activities are strongly influenced by the attitudes of the staff towards falls [21, 65]. In our study, we found that, despite the majority of staff acknowledging that falls are a serious problem in LTCFs, they believed their own facilities had fewer serious problems. This positive attitude towards their facilities was influenced by prior fall prevention training. Additionally, our results demonstrate that LTCF staff generally have high levels of confidence and motivation to participate in fall prevention activities.

Staff skills are essential to providing residents with the care and assistance they need, according to the National Standards for LTCF for Older People in Ireland, provided by Health Information and Quality Authority. Each staff member is required to complete relevant training that is suited to his/her position as part of a continuous professional development programme [66]. According to our findings, half of the respondents had previously received training on a range of fall prevention-related topics and resources. However, prior falls education or training appeared to have little impact on staff knowledge, noting that the timeframe for this prior education was up to five years previously, and that staff with extensive baseline falls knowledge may not have undertaken any recent education, while others with knowledge gaps may have undergone recent but limited (e.g., 1–2 h) education. Although nearly half the sample believed they had sufficient fall prevention education and training already, they indicated a preference for face-to-face education delivery in the workplace for any future education, consistent with earlier findings [33, 34].

Overall, we need to target the specific knowledge gaps to enhance knowledge of and competence in, and consequently behaviours linked to, fall-risk assessment and prevention, in order to develop more effective staff education interventions that would enhance present fall prevention practices in LTCFs [66]. These results highlight the need to offer customised fall prevention training that is suited to the particular learning needs of LTCF staff within a site, and considering the job roles of those attending. Given the observed knowledge gaps concerning falls, our findings might be utilised to inform future staff education programmes aimed at preventing falls in LTCFs.

### Limitations and strengths

This study provides important information on current staff attitudes, knowledge and confidence for fall prevention in LTCFs, and included various LTCF sizes and provider-types in both urban and rural settings, with 13 of 14 invited sites taking part. However, although the study targeted all LTCF staff, GPs and health and social care professionals had limited representation in the respondents. Although we offered two distribution methods for the survey (online/paper), our overall response rate was also low, possibly reflecting the coronavirus pandemic’s impact on LTCFs, with changed work practices and increased workload, along with LTCF staff having no protected time to take part in such surveys. Equally, the notable difference in response rates across sites (from 1 to 55%) indicates that site management and champion involvement, or site culture, may have influenced response rates. A low response rate in a site raises the possibility of responder bias, wherein those with good interest and knowledge of fall prevention may be more likely to complete the survey, and so data may not fully represent all staff, but rather those with an interest in this area. We have presented data for low-response and high-response sites which explored site-level differences, but it is not possible to present data about staff who did not respond. Notably, there was no difference in fall prevention knowledge between the respondents from high- and low-response sites, but there were differences in attitudes and confidence, which suggested that the respondents from low-response sites may have been particularly motivated and concerned about falls prevention.

This study used an already-existing fall knowledge test to measure staff knowledge levels, but the scoring rewards guessing and a strategy of ‘ticking all answers’, as there is no penalty for incorrect choices in the multiple-choice answers (e.g., if only one answer in a question is correct and the respondent ticks all four as correct, they receive the point, similar to the respondent who only selected the correct answer). Future research should examine the relationship between staff knowledge, attitudes and confidence, which was not analysed in this study.

## Conclusion

This study aimed to explore LTCF staff knowledge, attitudes and confidence about fall prevention among residents, to inform a future tailored educational intervention. LTCF staff in our sample overall have a high level of knowledge, positive attitude and confidence about fall prevention. More clinical years of experience working with older people in health care services was associated with greater fall knowledge and confidence, more so than self-reported prior fall prevention education. The findings indicate a need for role-specific educational interventions targeting certain knowledge gaps, such as the value of post-fall analyses and problem-solving techniques, fall risk factors (i.e., the usage of antipsychotic drugs) and fall prevention interventions (i.e., regular medication reviews). All staff require greater knowledge and training on fall prevention, but nurses and HCAs especially, and some will need to be motivated to undertake further education training, which should ideally be provided in person and on-site at their facilities to meet their stated preference. Future fall prevention education programmes should take into account the areas with greatest knowledge gaps, along with the groups that need this knowledge, and staff preferences regarding delivery. The particular challenge of providing multidisciplinary training to address all elements holistically, where the learner’s job role dictates both knowledge gaps and their potential to apply that knowledge, needs further consideration.

### Electronic supplementary material

Below is the link to the electronic supplementary material.

**Supplementary File 1**: The survey instrument

**Supplementary File 2**: The influence of demographic data on falls knowledge

**Supplementary file 3**: liner regression model to predict the variable effect of fall-knowledge test

**Supplementary 4**: Kruskal-Wallis test regarding staff confidence in their abilities to complete fall prevention activities to prevent residents from falling during their shift, categorised across their years of experience

**Supplementary file 5**: Previous training received and future training preferences

**Supplementary file 6**: Staff descriptions of the previous fall prevention training they underwent during the previous five years

## Data Availability

All dataset used and analysed during this study is available in from the corresponding author on a reasonable project.

## Abbreviations

LTCF: Long-term care facilities
WHO: The World Health Organisation
NICE: The National Institute for Clinical Excellence
HSE: The Health Service Executive
MDT: A multidisciplinary team
STROBE: The Strengthening the Reporting of Observational Studies in Epidemiology guidelines
DON: The director of nursing
GPs: General practitioners
HSCP: Health social care and professionals
COM-B: The Capability, Opportunity, and Motivation to Undertake a Health Behaviour Change framework
AHRQ: The Agency for Healthcare Research and Quality
HCAs: Health care assistants
FETAC: Further Education and Training Awards Council
SREC: The Social Research Ethics Committee
UCC: University College Cork
ADLs: The activities of daily livings
HIQA: Health information and quality authority
