# Supplementary material for: Staff knowledge, attitudes and confidence levels for fall preventions in older person long-term care facilities: a cross-sectional study
Source: BMC Geriatr. 2023 Sep 25;23:595. doi: 10.1186/s12877-023-04323-0 (PMC10521420; doi:10.1186/s12877-023-04323-0)
Supplement: Supplementary file 1 — The survey instrument [file 12877_2023_4323_MOESM1_ESM.docx]

Supplementary File 1: The survey instrument

| 1. 2E: Fall Knowledge Test (Ganz DA, 2013) | The modified questions / deleted questions/ added new questions |
| --- | --- |
| Each question may have more than one option as the correct answer.  Please circle the letters that correspond to the correct answers.     1. Which of the following statements is *correct*? 2. Falls have multifactorial etiology, so fall prevention programs should comprise multifaceted interventions. 3. Regular review of medication can help to prevent patient falls. 4. The risk of falling will be lessened when a patient’s toileting needs are met. 5. The use of antipsychotic medications is associated with an increased risk of falls in older adults.   2. A multifaceted intervention program should include:   1. Individually-tailored fall prevention strategies 2. Education to patient/family and health care workers 3. Environmental safety 4. Safe patient handling   3. Risk factors for falls in the acute hospital include all of the following *except*:   1. Dizziness/vertigo 2. Previous fall history 3. Antibiotic usage 4. Impaired mobility from stroke disease   4. Which of the following statements is *true*?   1. The cause of a fall is often an interaction between patient’s risk, the environment, and patient risk behavior. 2. Increase in hazardous environments increases the risk of falls. 3. The use of a patient identifier (e.g., identification bracelet) helps to highlight to staff those patients at risk for falls. 4. A fall risk assessment should include review of history of falls, mobility problems, medications, mental status, continence, and other patient risks.   5. Patients with impaired mobility should be:   1. Confined to bed 2. Encouraged to mobilize with assistance 3. Assisted with transfers 4. Referred for exercise program or prescription of walking aids as appropriate   6. The management of the acutely confused patient should include all of the following *except*:   1. Moving patients away from the nursing station 2. Involving family members to sit with the patient 3. Orienting patients to the hospital environment 4. Reinforcing activity limits to patients and their families   7. Which of the following statements is *false*?   1. Fall prevention efforts are solely the nurses’ responsibility. 2. A patient who is taking four or more oral medications is at risk for falling. 3. A patient who is taking psychotropic medication is at higher risk for falling. 4. Testing or treatment for osteoporosis should be considered in patients who are at high risk for falls and fractures.   8. In hospital settings, intervention programs should include:   1. Staff education on fall precautions 2. Provision and maintenance of mobility aids 3. Postfall analysis and problem-solving strategy 4. Bed alarms for all patients, regardless of risk   9. When assessing patients, which of the following statements is *false*?   1. All patients should be assessed for fall risk factors at admission, at a change in status, after a fall, and at regular intervals. 2. Medication review should be included in the assessment. 3. All patients should have their activities of daily living and mobility assessed. 4. Environmental assessment is not important in the hospital as it is all standardized.   10. Risk factors for falls include:   1. Parkinson’s disease 2. Incontinence 3. Previous history of falls 4. Delirium   11. Exercise programs for ambulatory older adults should:   1. Be very aggressive 2. Be unsupervised 3. Be ongoing 4. Include individualized strength and balance training   12. Which of the following statements on education in fall prevention is *false*?   1. Education programs should target primarily health care providers, patients, and caregivers. 2. Education programs for staff should include the importance of fall prevention, risk factors for falls, strategies to reduce falls, and transfer techniques. 3. Instruction on safe mobility, with emphasis on high-risk patients, should be provided to both patients and families. 4. Education should only be given at the start of the fall prevention program.   13. Which of the following is recommended to improve patient safety?   1. Locking wheeled furniture when it is stationary. 2. Having nonslip flooring. 3. Placing frequently used items (including call bell, telephone, and remote control) within reach of the patient 4. Rounding hourly to address patient needs | **Based on your current knowledge, answer as many as you know of the following questions. Each question may have more than one correct answer and** **so please tick all that apply.** *(Please tick the letters that correspond to the correct answers- e.g. if you think “a” is the only correct answer, tick “a”; if you think “c” and “d” are the correct answers, please tick both “c” and “d”).*  **Q1**- Which of the following statements is *correct*?   1. Falls have multifactorial etiology, so fall prevention programs should comprise multifaceted interventions. 2. Regular review of medication can help to prevent patient falls. 3. The risk of falling will be lessened when resident toileting needs are met. 4. The use of antipsychotic medications is associated with an increased risk of falls in older adults.   **Q2**- A multifaceted intervention program should include:   1. Individually-tailored fall prevention strategies 2. Education to resident /family and health care workers 3. Environmental safety 4. Safe patient handling   **Q3** - Risk factors for falls in the nursing home include all of the following ***except***:   1. Dizziness/vertigo 2. Previous fall history 3. Antibiotic usage 4. Impaired mobility from stroke disease   **Q4-** Which of the following statements is *true*?   1. The cause of a fall is often an interaction between resident risk, the environment, and patient risk behavior. 2. Increase in hazardous environments increases the risk of falls. 3. The use of a patient identifier (e.g., identification bracelet) helps to highlight to staff those residents at risk for falls. 4. A fall risk assessment should include review of history of falls, mobility problems, medications, mental status, continence, and other resident risks.   **Q5-** Residents with impaired mobility should be:   1. Confined to bed 2. Encouraged to mobilize with assistance 3. Assisted with transfers 4. Referred for exercise program or prescription of walking aids as appropriate   **Q6-** The management of the acutely confused resident should include all of the following ***except*:**   1. Moving resident away from the nursing station 2. Involving family members to sit with the patient 3. Orienting residents to the nursing environment 4. Reinforcing activity limits to residents and their families   **Q7-** Which of the following statements is ***false***?   1. Fall prevention efforts are solely the nurses’ responsibility. 2. A resident who is taking four or more oral medications is at risk for falling. 3. A resident who is taking psychotropic medication is at higher risk for falling. 4. Testing or treatment for osteoporosis should be considered in residents who are at high risk for falls and fractures.   **Q8**- In long-term care settings, intervention programs should include:   1. Staff education on fall precautions 2. Provision and maintenance of mobility aids 3. Post fall analysis and problem-solving strategy 4. Bed alarms for all resident, regardless of risk   **Q9-** When assessing resident, which of the following statements is ***false***?   1. All residents should be assessed for fall risk factors at admission, at a change in status, after a fall, and at regular intervals. 2. Medication review should be included in the assessment. 3. All residents should have their activities of daily living and mobility assessed. 4. Environmental assessment is not important in the nursing home as it is all standardized.   **Q10-** Risk factors for falls include:   1. Parkinson’s disease 2. Incontinence 3. Previous history of falls 4. Delirium   **Q11-** Exercise programs for ambulatory older adults should:   1. Be very aggressive 2. Be unsupervised 3. Be ongoing 4. Include individualized strength and balance training   **Q12-** Which of the following statements on education in fall prevention is ***false***?   1. Education programs should target primarily health care providers, resident, and caregivers. 2. Education programs for staff should include the importance of fall prevention, risk factors for falls, strategies to reduce falls, and transfer techniques. 3. Instruction on safe mobility, with emphasis on high-risk residents, should be provided to both residents and families. 4. Education should only be given at the start of the fall prevention program.   **Q13**- Which of the following is recommended to improve resident safety?   1. Locking wheeled furniture when it is stationary. 2. Having nonslip flooring. 3. Placing frequently used items (including call bell, telephone, and remote control) within reach of the resident 4. Rounding hourly to address residents need |
| 1. COM-B survey (Hang et al., 2016) | **Modification of this study survey** |
| **Section 1 – Your details**   1. Please state your gender: (*Please tick* 🗹 *one)*   🞏 Male  🞏Female | **Section 1 – Can you tell us about yourself?**  **Q1**- Please state your gender:   1. Male 2. Female   3. Other 4. Prefer not to say |
| 1. Which age group describes you based on your last birthday? (*Please tick* 🗹 *one)*   🞏 18-19 years  🞏 20-29 years  🞏 30-39 years  🞏 40-49 years  🞏 50-59 years  🞏 60-65 years | **Q2**- Which age group describes you based on your last birthday?   1. 18-29 years 2. 30-39 years   3. 40-49 years 4. 50-59 years  5. 60-65 years 6. Prefer not to say |
| 1. What is the highest level of learning you have done? (*Please tick* 🗹 *one)*   🞏 Left school before Year 10  🞏 Completed Year 10  🞏 Completed Year 12  🞏 TAFE (Certificate I to IV) (Please specify: _________________________)  🞏 Graduate Certificate (Please specify: _________________________)  🞏 Graduate Diploma (Please specify: _________________________)  🞏 Bachelor degree (Please specify: _________________________)  🞏 Master degree (Please specify: _________________________)  🞏 Other and / or overseas (Please specify: _________________________) | **Q3**- What is the highest level of learning you have done?   1. FETAC level 3/4/5/6 (Please specify : _________________________) 2. Post Graduate Certificate (Please specify: _________________________) 3. Post Graduate Diploma (Please specify: _________________________) 4. Bachelor degree (Please specify: _________________________) 5. Master degree (Please specify: _________________________) 6. Other (Please specify: _________________________) |
| 1. How long have you worked as a carer for older people either at this organisation or somewhere else? (*Please tick* 🗹 *one)*   🞏 more than 3 months but less than 6 months  🞏 6-12 months  🞏 1-2 years  🞏 3-5 years  🞏 6-10 years  🞏 more than 11 years | **Q4**- How long have you worked as a career for older people in total either at this organisation or somewhere else?   1. Less than one year 2. 1-2 years 3. 3-5 years 4. 6-10 years 5. more than 11 years |
| 1. How long have you worked at this organisation? (*Please tick* 🗹 *one)*   🞏 less than 6 months  🞏 6-12 months  🞏 1-2 years  🞏 3-5 years  🞏 6-10 years  🞏 more than 11 years | **Q5**- How long have you worked in this residential care facility?   1. 3-6 months 2. 7-12 months   3. 1-2 years 4. 3-5 years  5. 6-10 years 6. More than 11 years |
| 1. What level(s) of care are you involved in delivering for the residents?   (*Please tick* 🗹 *all that apply)*  🞏 High level care  🞏 Low level care  🞏 Dementia specific care  🞏 Unsure | ***Excluded this Question***  we added a new question*  **Q6**- What is your role in this residential care facility?*   1. Nurse 2. Senior Nurse/CNM 3. Health Care Assistant 4. GP 5. HSCP 6. Other (Please specify: _________________________) |
| 1. What shift(s) do you work? (*Please tick* 🗹 *all that apply)*   🞏 Morning  🞏 Afternoon  🞏 Night | **Q7**- What shift(s) do you work? Tick all that apply. (Please skip this question if you are a GP)   1. Morning 2. Afternoon 3. Full Day (12 hours) 4. Night (12 hours) 5. Twilight hours |
| 1. What language do you mainly speak at home? (*Please tick* 🗹 *one)*   🞏 English (please go to question 10)  🞏 Other (Please specify: ______________________________________) | ***Excluded this question*** |
| 1. If you speak a language other than English, do you have any problem writing, reading or speaking in English? (*Please tick* 🗹 *one)*   🞏 Yes 🞏 Reading 🞏 No  🞏 Writing  🞏 Speaking | ***Excluded this question*** |
| **Section 2 – This section asks for your feedback about falls or near falls and possible injuries that residents may experience** | **Section 2a: Please answer the following questions about falls prevention** |
| 1. How would you describe “a fall” in your own words? | ***Excluded this question*** |
| 1. Do you think resident’s falls can be prevented from happening?   (*Please tick* 🗹 *one)*  🞏 Yes 🞏 No 🞏 Unsure | Excluded this question |
| 1. Have you done any training to help prevent falls in the past? (*Please tick* 🗹 *one)*   🞏 Yes 🞏 No 🞏 Unsure | **Q21-** Have you completed any training in fall prevention in the last five years?   1. Yes 2. No 3. Unsure |
| 1. If you answered “yes” in question 12, please tell us a little bit about the training.   ________________________________________________________________________________________________________________________________ | **Q22-** If you answered “yes” in question 21, please tell us a little bit about the training. If not, please skip to Question 23.   1. Length of the training (hours or days) _______ 2. Who delivered it___________________________________ 3. Location of training (e.g., onsite, in a university or hotel) ___________ 4. Resources provided (e.g., manual, book, etc) ________________________ 5. Any other comments on the training _______________________________ |
| 1. List the things you think could help prevent residents from falling.   ________________________________________________________________________________________________________________________________  ________________________________________________________________ | **Q23-** List the top 3 things you think could help your site to better prevent residents from falling.  1._____________________________________________________________________________  2.______________________________________________________________________  3._____________________________________________________________________________ |
| 1. List any things you think could prevent residents **injuring** themselves if they fall. | **Q24-** List the top 3 things you think could prevent residents from **injuring** themselves if they fall.  1.___________________________________________________________________________  2.____________________________________________________________________________  3.____________________________________________________________________________ |
|  |  |
| ____________________________________________________________ |  |
| 1. When thinking of all the residents at your site (as a group), would you say they were: (*Please tick* 🗹 *one)*   🞏 At very high risk of falls  🞏 At moderate risk of falls  🞏 At low risk of falls  🞏 Unsure | ***Excluded this question*** |
| **Section 3 – This section asks about how you think about falls prevention when you are completing your shifts. Please read the following statements and rate your response.** | **Section 3 – This section asks about how you think about your own role in fall prevention. Please read the following statements and choose the option that best reflects your opinion.** |
| 1. When working my rostered shift, I feel confident that I know what to do to prevent residents from falling. (*Please tick* 🗹 *one)*   🞏 Strongly agree 🞏 Agree 🞏 Undecided  🞏 Disagree 🞏 Strongly disagree | **Q25-** When working my rostered shift, I feel confident that I know what to do to prevent residents from falling.   1. Strongly agree 2. Agree 3. Undecided 4. Disagree 5. Strongly disagree |
| 1. When working my rostered shift, I am keen to prevent residents from falling.   (*Please tick* 🗹 *one)*  🞏 Strongly agree 🞏 Agree 🞏 Undecided  🞏 Disagree 🞏 Strongly disagree | **Q26-** When working my rostered shift, I am keen to prevent residents from falling.   1. Strongly agree 2. Agree 3. Undecided 4. Disagree 5. Strongly disagree |
| 1. When working my rostered shift, I am confident that I can complete actions that can prevent residents from falling. (*Please tick* 🗹 *one)*   🞏 Strongly agree 🞏 Agree 🞏 Undecided  🞏 Disagree 🞏 Strongly disagree | **Q27-** When working my rostered shift, I am confident that I can complete actions that can prevent residents from falling.   1. Strongly agree 2. Agree 3. Undecided 4. Disagree 5. Strongly disagree |
| 1. What percentage of older people do you think fall in residential aged care homes every year? (*Please tick* 🗹 *one)*   🞏 10% 🞏 20% 🞏 50% | **Q28-** What percentage of older people do you think fall in residential aged care homes every year?  1- 10% 2- 20% 3- 50% 4- Unsure |
| 1. What would you do if a resident has fallen over during your shift? Briefly describe the actions you would take. | **Q29-** What would you do if a resident had fallen during your shift? Briefly list the actions you would take. |
| 1. Do you get any information at work on how to prevent residents from having a fall? (*Please tick* 🗹 *one)*   🞏 Yes 🞏 No 🞏 Unsure | **Q30-** Do you get any information at work on how to prevent residents from having a fall?   1. Yes 2. No 3. Unsure |
| 1. Is there a falls prevention plan in the notes of the residents you are currently working with? (*Please tick* 🗹 *one)*   🞏 Yes (Answer Q. 24) 🞏 No (Go to Q. 25) 🞏 Unsure (Go to Q. 25) | **Q31-** Is there a falls prevention plan in the notes of the residents you are currently working with?   1. Yes 2. No 3. Unsure |
| 1. If you answered Yes to the question 23, could you tell us a bit about the plan to help you stop residents you care for falling? | **Q32-** If you answered Yes to the question 32, could you tell us a bit about the plan to help you stop residents you care for falling? Otherwise please move to the Question 34. |
| 1. Do you share information with other care staff at work about how to prevent falls for the residents you care for? (*Please tick* 🗹 *one)*   🞏 Yes 🞏 No 🞏 Unsure | ***Excluded this question*** |
| 1. I work as part of a team (nurses, manager, physiotherapist, other organisational staff at facility) to prevent falls in my work place (*Please tick* 🗹 *one)*   🞏 Strongly agree 🞏 Agree 🞏 Undecided  🞏 Disagree 🞏 Strongly disagree | ***Excluded this question*** |
| 1. I think falls are a serious problem in residential aged care homes. (*Please tick* 🗹 *one)*   🞏 Strongly agree 🞏 Agree 🞏 Undecided  🞏 Disagree 🞏 Strongly disagree | **Q33-** I think falls are a serious problem in residential aged care homes.   1. Strongly agree 2. Agree 3. Undecided 4. Disagree 5. Strongly disagree |
| 1. I think falls are a serious problem across this organisation. (*Please tick* 🗹 *one)*   🞏 Strongly agree 🞏 Agree 🞏 Undecided  🞏 Disagree 🞏 Strongly disagree | **Q34-** I think falls are a serious problem in this facility.   1. Strongly agree 2. Agree 3. Undecided 4. Disagree 5. Strongly disagree |
| **Section 4 – This section asks you about how you think falls prevention training could be provided to care staff in this organisation** | **Section 4 – This section asks you about how falls prevention training could be provided to care staff in this facility** |
| 1. I think I have already had enough training about how to prevent falls. (*Please tick* 🗹 *one)*   🞏 Strongly agree 🞏 Agree 🞏 Undecided  🞏 Disagree 🞏 Strongly disagree | **Q35-** I think I have already had enough training about how to prevent falls.   1. Strongly agree 2. Agree 3. Undecided 4. Disagree 5. Strongly disagree |
| 1. If the organisation gave care staff training on preventing falls in the future, would you like training to be: (*Please tick* 🗹 *one)*   🞏 E-learning (using a computer to watch, read and comment on falls and falls prevention)  🞏 Watching a DVD on falls and falls prevention  🞏 Attending an ‘In-service’ training session on falls and falls prevention including listening to a talk, watching some video clips and having a discussion | **Q36-** If the organisation gave care staff training on preventing falls in the future, would you like training to be:   1. E-learning (using a computer to watch, read and comment on falls and falls prevention) 2. Watching a DVD on falls and falls prevention (with no interaction) 3. Attending an ‘In-service’ training session on falls and falls prevention including listening to a talk, watching some video clips and having a discussion |
| 1. Where would you like to attend training on preventing falls? (*Please tick* 🗹 *one)*   🞏 Organisation’s central training centre  🞏 Your facility  🞏 No preference | ***Excluded this question*** |
| 1. Would you like reminders to help you know and use actions to prevent falls when you are at work? (*Please tick* 🗹 *one)*   🞏 Yes 🞏 No 🞏 Unsure | ***Excluded this question*** |
| 1. If you answered Yes to question 32, what type(s) of reminder would you like? (*Please tick* 🗹 *one)*   🞏 Written checklist in resident file  🞏 Picture/photographic checklist in resident file  🞏 Written checklist on the back of my name badge  🞏 Posters around facility | ***Excluded this question*** |
| 1. What language(s) would you like the information on preventing falls to be available in? Please specify | ***Excluded this question*** |
| 1. What do you think could make it difficult to carry out falls prevention actions during your shift? | ***Excluded this question*** |
| 1. Please tell us anything else you think would help make this questionnaire easier for other care staff to answer. | ***Excluded this question*** |
|  | **New questions added *** |
|  | **Q37-** What would help you use training you have received **in practice** to prevent falls when you are at work?*  **Q 38-** Are there any other comments you would like to add about falls prevention in residential care facilities?* |
| Underlined text indicates modified questions or wording from the original surveys; bold, underlined and italicized text indicates questions that were excluded; and * items and underlined denotes the new questions added. | |

**References:**

Ganz DA, H. C. S. D. et a . A. for H. R. Agency. 2EFall K. T. (2013). Preventing Falls in Hospitals: A Toolkit for Improving Quality of Care. *Agency for Healthcare Research and Quality*, *13*(0015-EF:199-22). www.ahrq.gov

Hang, J. A., Francis-Coad, J., Burro, B., Nobre, D., & Hill, A. M. (2016). Assessing knowledge, motivation and perceptions about falls prevention among care staff in a residential aged care setting. *Geriatric Nursing*, *37*(6), 464–469. https://doi.org/10.1016/j.gerinurse.2016.06.019
