# Supplementary figures and images for: Staff knowledge, attitudes and confidence levels for fall preventions in older person long-term care facilities: a cross-sectional study
Source: BMC Geriatr. 2023 Sep 25;23:595. doi: 10.1186/s12877-023-04323-0 (PMC10521420; doi:10.1186/s12877-023-04323-0)

**Supplementary File 2: The influence of demographic data on falls knowledge**

| 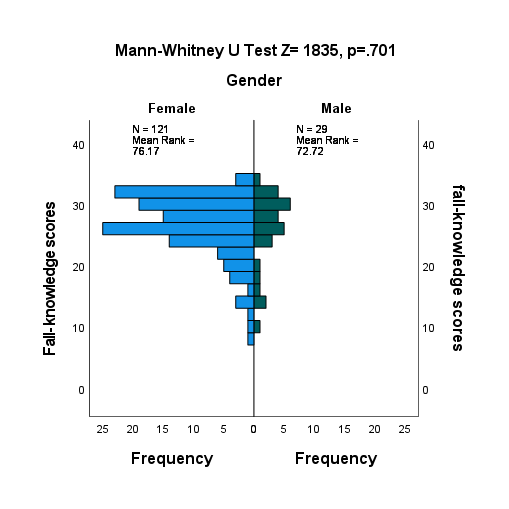 | 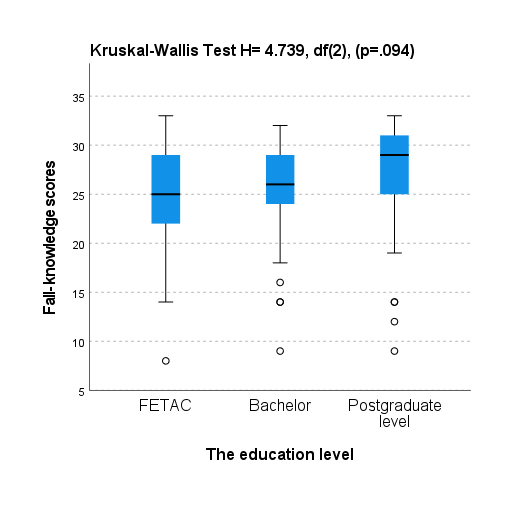 |
| --- | --- |
| 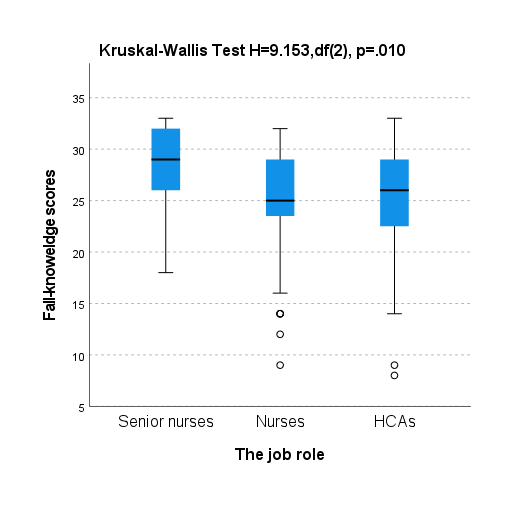 | 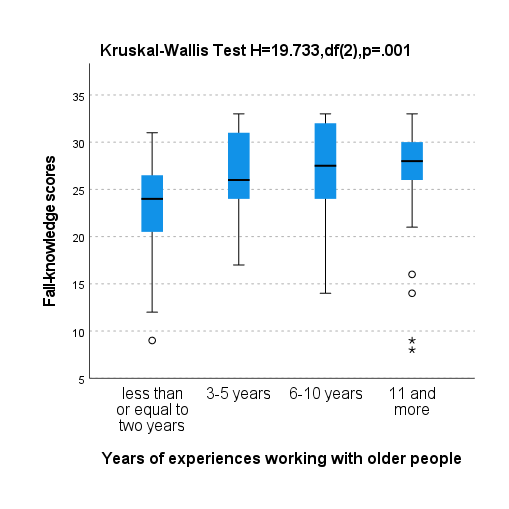 |
| 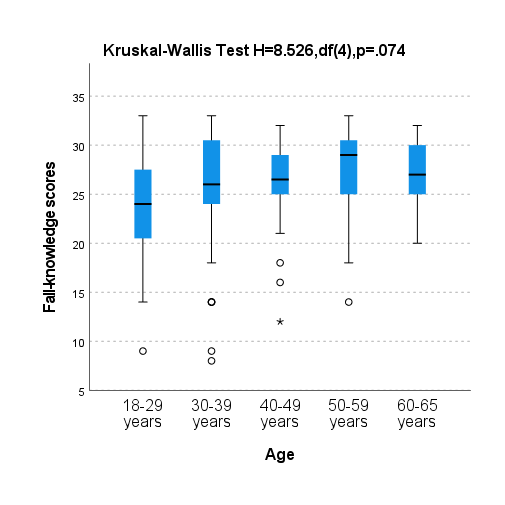 | 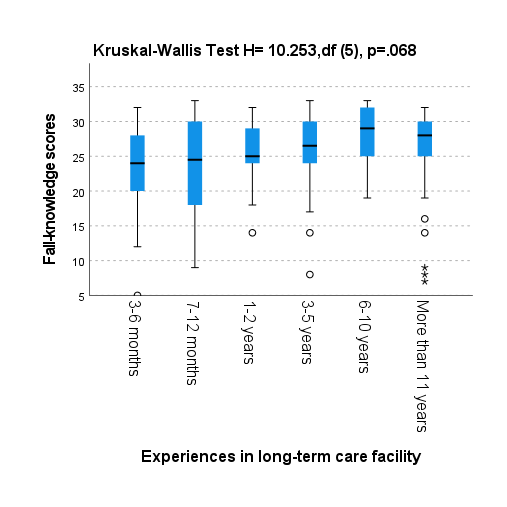 |
| 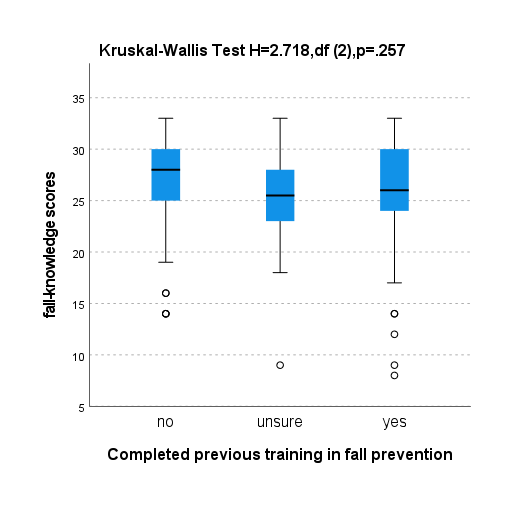 |  |

Supplement: Supplementary file 2 — The influence of demographic data on falls knowledge [file 12877_2023_4323_MOESM2_ESM.docx]
