# Supplementary material for: Staff knowledge, attitudes and confidence levels for fall preventions in older person long-term care facilities: a cross-sectional study
Source: BMC Geriatr. 2023 Sep 25;23:595. doi: 10.1186/s12877-023-04323-0 (PMC10521420; doi:10.1186/s12877-023-04323-0)
Supplement: Supplementary file 3 — liner regression model to predict the variable effect of fall-knowledge test [file 12877_2023_4323_MOESM3_ESM.docx]

Supplementary file 3: liner regression model to predict the variable effect of fall-knowledge test

| Residuals before transformation | Residual transformation by Log 10 |
| --- | --- |
| 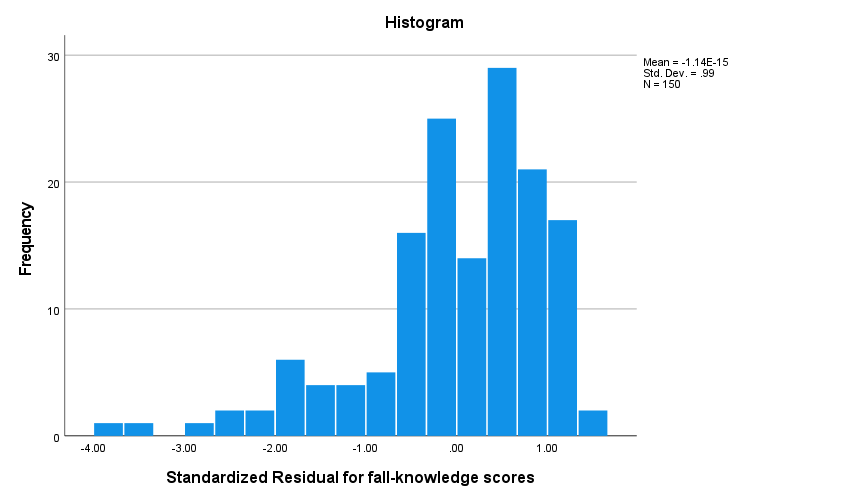 | 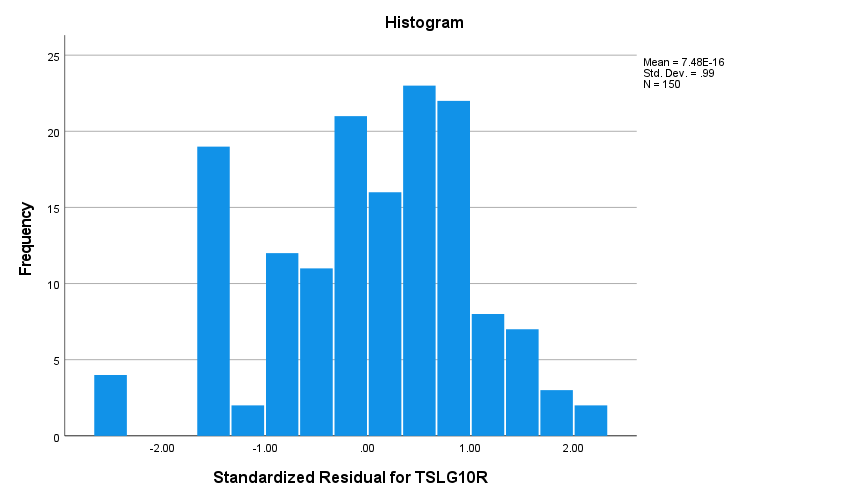 |

| \| **Tests of Between-Subjects Effects of linear regression** \| \| \| \| \| \| \| --- \| --- \| --- \| --- \| --- \| --- \| \| Dependent Variable: TSLG10R of fall- knowledge scores \| \| \| \| \| \| \| Source \| Type III Sum of Squares \| df \| Mean Square \| F \| Sig. \| \| Corrected Model \| 1.942^a^ \| 8 \| .243 \| 2.560 \| .013 \| \| Intercept \| 39.960 \| 1 \| 39.960 \| 421.258 \| <.001 \| \| Years experiences \| 1.464 \| 3 \| .488 \| 5.146 \| .002 \| \| Gender \| .000 \| 1 \| .000 \| .002 \| .965 \| \| Education levels \| .156 \| 2 \| .078 \| .820 \| .443 \| \| Previous training in falls prevention \| .097 \| 2 \| .049 \| .513 \| .600 \| \| Error \| 12.711 \| 134 \| .095 \|  \|  \| \| Total \| 106.602 \| 143 \|  \|  \|  \| \| Corrected Total \| 14.654 \| 142 \|  \|  \|  \| \| a. R Squared = .133 (Adjusted R Squared = .081) \| \| \| \| \| \| |
| --- | --- | --- | --- | --- | --- | --- | --- | --- | --- | --- | --- | --- | --- | --- | --- | --- | --- | --- | --- | --- | --- | --- | --- | --- | --- | --- | --- | --- | --- | --- | --- | --- | --- | --- | --- | --- | --- | --- | --- | --- | --- | --- | --- | --- | --- | --- | --- | --- | --- | --- | --- | --- | --- | --- | --- | --- | --- | --- | --- | --- | --- | --- | --- | --- | --- | --- | --- | --- | --- | --- | --- | --- | --- | --- | --- | --- | --- | --- |
| \| **Tests of Between-Subjects Effects with elimination gender** \| \| \| \| \| \| \| --- \| --- \| --- \| --- \| --- \| --- \| \| Dependent Variable: TSLG10R of fall- knowledge scores \| \| \| \| \| \| \| Source \| Type III Sum of Squares \| df \| Mean Square \| F \| Sig. \| \| Corrected Model \| 2.143^a^ \| 7 \| .306 \| 3.285 \| .003 \| \| Intercept \| 57.213 \| 1 \| 57.213 \| 613.965 \| <.001 \| \| Years experiences \| 1.687 \| 3 \| .562 \| 6.033 \| <.001 \| \| Education levels \| .166 \| 2 \| .083 \| .890 \| .413 \| \| Previous training in falls prevention \| .104 \| 2 \| .052 \| .557 \| .574 \| \| Error \| 12.766 \| 137 \| .093 \|  \|  \| \| Total \| 109.292 \| 145 \|  \|  \|  \| \| Corrected Total \| 14.909 \| 144 \|  \|  \|  \| \| a. R Squared = .144 (Adjusted R Squared = .100) \| \| \| \| \| \| |
| \| **Tests of Between-Subjects Effects with elimination previous training** \| \| \| \| \| \| \| --- \| --- \| --- \| --- \| --- \| --- \| \| Dependent Variable: TSLG10R of fall- knowledge scores \| \| \| \| \| \| \| Source \| Type III Sum of Squares \| df \| Mean Square \| F \| Sig. \| \| Corrected Model \| 2.039^a^ \| 5 \| .408 \| 4.404 \| <.001 \| \| Intercept \| 67.752 \| 1 \| 67.752 \| 731.734 \| <.001 \| \| Years experiences \| 1.669 \| 3 \| .556 \| 6.008 \| <.001 \| \| Education levels \| .230 \| 2 \| .115 \| 1.239 \| .293 \| \| Error \| 12.870 \| 139 \| .093 \|  \|  \| \| Total \| 109.292 \| 145 \|  \|  \|  \| \| Corrected Total \| 14.909 \| 144 \|  \|  \|  \| \| a. R Squared = .137 (Adjusted R Squared = .106) \| \| \| \| \| \| |
| \| **Tests of Between-Subjects Effects with elimination education levels** \| \| \| \| \| \| \| --- \| --- \| --- \| --- \| --- \| --- \| \| Dependent Variable: TSLG10R \| \| \| \| \| \| \| Source \| Type III Sum of Squares \| df \| Mean Square \| F \| Sig. \| \| Corrected Model \| 1.800^a^ \| 3 \| .600 \| 6.644 \| <.001 \| \| Intercept \| 74.623 \| 1 \| 74.623 \| 826.317 \| <.001 \| \| Years of experiences \| 1.800 \| 3 \| .600 \| 6.644 \| <.001 \| \| Error \| 13.185 \| 146 \| .090 \|  \|  \| \| Total \| 112.906 \| 150 \|  \|  \|  \| \| Corrected Total \| 14.985 \| 149 \|  \|  \|  \| \| a. R Squared = .120 (Adjusted R Squared = .102) \| \| \| \| \| \| |
| \| **Multiple Comparisons (post hoc analysis)** \| \| \| \| \| \| \| \| --- \| --- \| --- \| --- \| --- \| --- \| --- \| \| (I) Years of experiences \| (J) Years of experiences \| Mean Difference (I-J) \| Std. Error \| Sig. \| 95% Confidence Interval \| \| \| Lower Bound \| Upper Bound \| \| less than or equal to two years \| 3-5 years \| .2412^*^ \| .07732 \| .013 \| .0344 \| .4480 \| \| 6-10 years \| .2273^*^ \| .08408 \| .046 \| .0024 \| .4522 \| \| 11 and more \| .2437^*^ \| .05867 \| <.001 \| .0868 \| .4006 \| \| 3-5 years \| less than or equal to two years \| -.2412^*^ \| .07732 \| .013 \| -.4480 \| -.0344 \| \| 6-10 years \| -.0139 \| .09457 \| 1.000 \| -.2669 \| .2390 \| \| 11 and more \| .0025 \| .07291 \| 1.000 \| -.1926 \| .1975 \| \| 6-10 years \| less than or equal to two years \| -.2273^*^ \| .08408 \| .046 \| -.4522 \| -.0024 \| \| 3-5 years \| .0139 \| .09457 \| 1.000 \| -.2390 \| .2669 \| \| 11 and more \| .0164 \| .08004 \| 1.000 \| -.1977 \| .2305 \| \| 11 and more \| less than or equal to two years \| -.2437^*^ \| .05867 \| <.001 \| -.4006 \| -.0868 \| \| 3-5 years \| -.0025 \| .07291 \| 1.000 \| -.1975 \| .1926 \| \| 6-10 years \| -.0164 \| .08004 \| 1.000 \| -.2305 \| .1977 \| \| Based on observed means.  The error term is Mean Square(Error) = .090. \| \| \| \| \| \| \| \| *. The mean difference is significant at the .05 level. \| \| \| \| \| \| \| |

| **Correlations** | | | | | |
| --- | --- | --- | --- | --- | --- |
|  | | Education level | The job role | Years of experiences | Gender |
| Education level | Pearson Correlation | 1 | -.579^**^ | .188^*^ | .003 |
|  | Sig. (2-tailed) |  | <.001 | .024 | .971 |
|  | N | 146 | 131 | 145 | 144 |
| The job role | Pearson Correlation | -.579^**^ | 1 | -.255^**^ | -.274^**^ |
|  | Sig. (2-tailed) | <.001 |  | .003 | .001 |
|  | N | 131 | 135 | 135 | 133 |
| Years of experiences | Pearson Correlation | .188^*^ | -.255^**^ | 1 | .103 |
|  | Sig. (2-tailed) | .024 | .003 |  | .213 |
|  | N | 145 | 135 | 150 | 148 |
| Gender | Pearson Correlation | .003 | -.274^**^ | .103 | 1 |
|  | Sig. (2-tailed) | .971 | .001 | .213 |  |
|  | N | 144 | 133 | 148 | 150 |
| **. Correlation is significant at the 0.01 level (2-tailed). | | | | | |
| *. Correlation is significant at the 0.05 level (2-tailed). | | | | | |
