# Supplementary material for: Staff knowledge, attitudes and confidence levels for fall preventions in older person long-term care facilities: a cross-sectional study
Source: BMC Geriatr. 2023 Sep 25;23:595. doi: 10.1186/s12877-023-04323-0 (PMC10521420; doi:10.1186/s12877-023-04323-0)
Supplement: Supplementary file 4 — Supplementary 4: Kruskal-Wallis test regarding staff confidence in their abilities to complete fall prevention activities to prevent residents from falling during their shift, categorised across their years of experience [file 12877_2023_4323_MOESM4_ESM.docx]

Supplementary 4: Kruskal-Wallis test regarding staff confidence in their abilities to complete fall prevention activities to prevent residents from falling during their shift, categorised across their years of experience

| **Pairwise Comparisons of Years of experience** | | | | | |
| --- | --- | --- | --- | --- | --- |
|  | Test Statistic | Std. Error | Std. Test Statistic | Sig. | Adj. Sig. ^a^ |
| 3-5 years versus 11 years and more | -4.806 | 9.327 | -.515 | .606 | 1.000 |
| 3-5 years versus Two years or less | 16.461 | 9.777 | 1.684 | .092 | .554 |
| 3-5 years versus 6-10 years | -34.167 | 11.945 | -2.860 | .004 | .025 |
| 11 years and more versus Two years or less | 11.655 | 7.360 | 1.583 | .113 | .680 |
| 11 years and more versus 6-10 years | 29.360 | 10.063 | 2.918 | .004 | .021 |
| Two years or less versus 6-10 years | -17.706 | 10.482 | -1.689 | .091 | .547 |
| \| Each row tests the null hypothesis that the Sample 1 and Sample 2 distributions are the same.  Asymptotic significances (2-sided tests) are displayed. The significance level is .050. \| \| --- \| \| a. Significance values adjusted using the Bonferroni correction for multiple tests. \| | | | | | |

Kruskal-Wallis Test H: 11.351, df(3),p=.010
