# Supplementary material for: Staff knowledge, attitudes and confidence levels for fall preventions in older person long-term care facilities: a cross-sectional study
Source: BMC Geriatr. 2023 Sep 25;23:595. doi: 10.1186/s12877-023-04323-0 (PMC10521420; doi:10.1186/s12877-023-04323-0)
Supplement: Supplementary file 5 — Previous training received and future training preferences [file 12877_2023_4323_MOESM5_ESM.docx]

Supplementary file 5: Previous training received and future training preferences

| Education training for fall prevention and educational preferences of staff | | | N | % |
| --- | --- | --- | --- | --- |
| Completed any training in fall prevention in the last five years | No | | 52 | 33.5 |
|  | Unsure | | 24 | 15.5 |
|  | Yes | | 78 | 50.3 |
|  | Missing | | 1 | 0.6 |
| I think I have already had enough training about how to prevent falls | Strongly Disagree | | 4 | 2.6 |
|  | Disagree | | 37 | 23.9 |
|  | Undecided | | 33 | 21.3 |
|  | Agree | | 50 | 32.3 |
|  | Strongly agree | | 22 | 14.2 |
|  | Missing | | 9 | 5.8 |
| Future preferences for training delivery modes | One training delivery mode selected | E-learning (n=30) | 113 | 72.9 |
|  |  | Watching DVD (n=2) |  |  |
|  |  | In-service (n=81) |  |  |
|  | Two training delivery modes selected | E-learning and in-service (n=13) | 19 | 12.3 |
|  |  | E-learning and DVD (n=2) |  |  |
|  |  | In-service and DVD (n=4) |  |  |
|  | Three training delivery modes | | 8 | 5.2 |
|  | Missing | | 15 | 9.7 |
