# Supplementary material for: Staff knowledge, attitudes and confidence levels for fall preventions in older person long-term care facilities: a cross-sectional study
Source: BMC Geriatr. 2023 Sep 25;23:595. doi: 10.1186/s12877-023-04323-0 (PMC10521420; doi:10.1186/s12877-023-04323-0)
Supplement: Supplementary file 6 — Staff descriptions of the previous fall prevention training they underwent during the previous five years [file 12877_2023_4323_MOESM6_ESM.docx]

Supplementary file 6: Staff descriptions of the previous fall prevention training they underwent during the previous five years

| Training parameters | Details | | Total responses: 70 |
| --- | --- | --- | --- |
| The length of training | - 35 healthcare workers attended training for various hours, ranging from **an hour to 5-6** hours per day - 8 healthcare workers attended a full-day training - 4 healthcare workers had ongoing annual training | | 47 |
| Location of training | - 38 healthcare workers attended **face-to-face** on-site training | - Mercy University Hospital (n=1) | 59 |
|  |  | - St. Luke’s Hospital (n=2) |  |
|  |  | - Northridge Education Center (n=4) |  |
|  |  | - Ballincollig Care Unit (n=1) |  |
|  |  | - In-service (n=30) |  |
|  | - 20 attended an online training | - **Health Service Executive** (HSE) training online(n=9) |  |
|  | - 1 attended a conference related to fall prevention | |  |
| Resources provided during training and the educational materials provided | - 12 had handed out manual resources - 4 had provided online resources - 2 had manual and online resources - One stated that no resources were supplied | - **Slide presentation** (n = 5) - Lectures (n = 4) - Information sheet (n = 3) - Reading resources (n = 1) - Videos (n = 1) | 19 |
| Topics that were covered in the training | - **Fall prevention** education/training sessions and update information (n= 9) - Fall **risk assessment** (n = 5) - Manual **handling techniques** (n = 4) - Having a course and modules related to fall prevention (n=3): ISCP, Parkinson’s association and MS society; health and safety modules; health care support level 5 course - Fall education involved residents’ education (n = 2) - Environmental safety (n = 1) | | 21 |
| Speakers who delivered the training | - PT and/or OT (n = 7) - Nurses, Director of nursing, and assistant director of nursing (n = 5) - External training providers (n = 2) | | 14 |
